# Supplementary material for: Properties of Poly(3-hydroxybutyrate-co-3-hydroxyvalerate)/Polycaprolactone Polymer Mixtures Reinforced by Cellulose Nanocrystals: Experimental and Simulation Studies
Source: Polymers (Basel). 2022 Jan 16;14(2):340. doi: 10.3390/polym14020340 (PMC8780583; doi:10.3390/polym14020340)
Supplement: Supplementary file 1 [file polymers-14-00340-s001.zip › polymers-1531469-supplementary.pdf]

Supplementary Material

Properties of Poly(3-hydroxybutyrate-*co*-3-hydroxyvalerate)/ Polycaprolactone Polymer Mixtures Reinforced by Cellulose Nanocrystals: Experimental and Simulation Studies

Marina I. Voronova, Darya L. Gurina and Oleg V. Surov*

G.A. Krestov Institute of Solution Chemistry of the Russian Academy of Sciences; miv@isc-ras.ru (M.V.), gdl@isc-ras.ru (D.G.)

* Correspondence: ovs@isc-ras.ru; Tel.: (+7-493-2351-545)

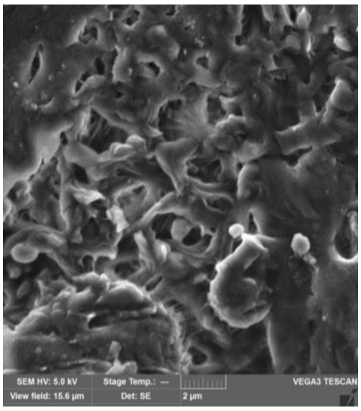

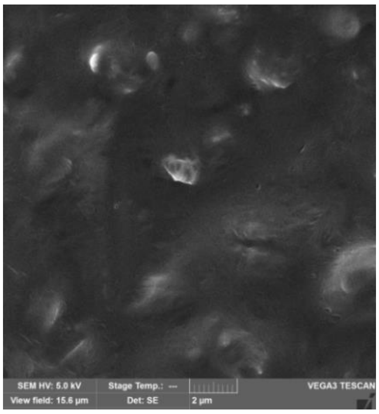

(**a**) (**b**)

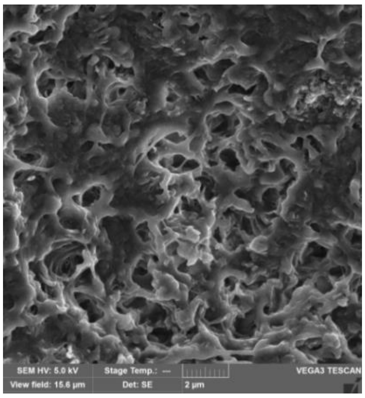

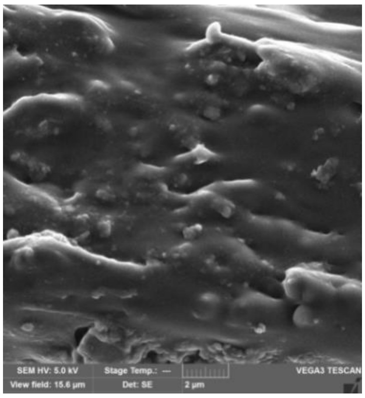

(**c**) (**d**)

Figure S1. SEM images of the surface (**a**, **b**) and cleavage (**c**, **d**) of the PHBV/PCL mixture films: 1:1 (**a**, **c**); 1:2 (**b**, **d**). The scale is 2 μm.

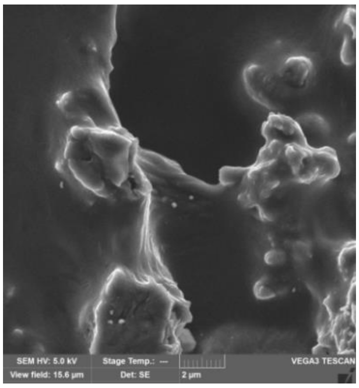

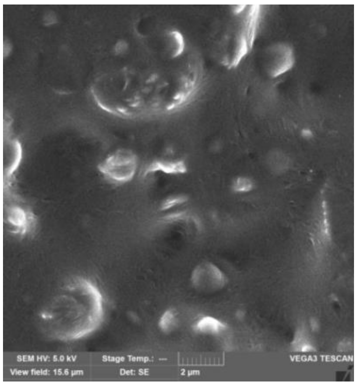

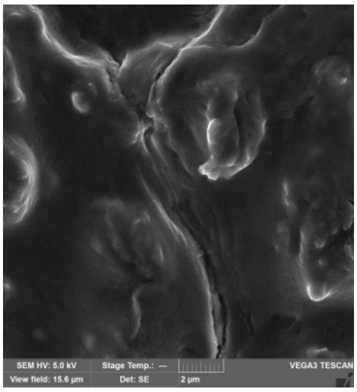

(**a**) (**b**) (**c**)

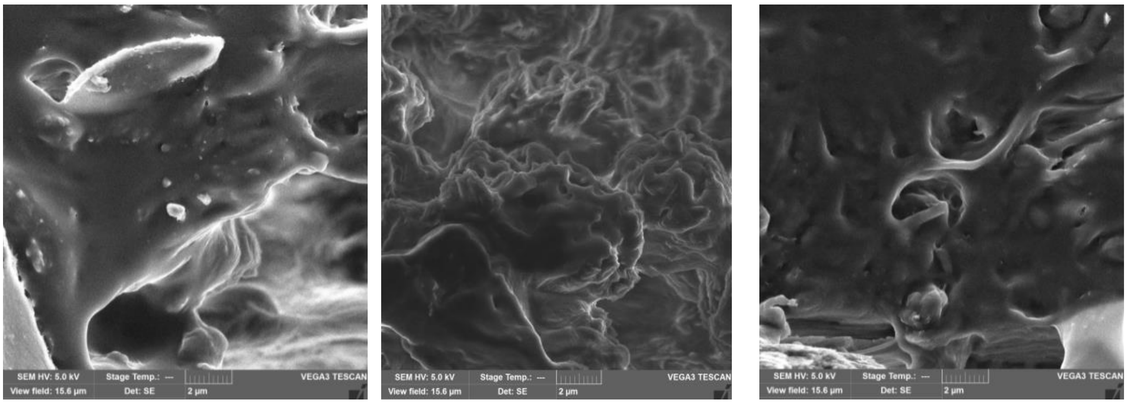

(**d**) (**e**) (**f**)

Figure S2. SEM images of the surface (**a**-**c**) and cleavage (**d**-**f**) of the PHBV/PCL/CNC composite films: PHBV/PCL(1:2)/CNC(PVP)-5 (**a**, **d**); PHBV/PCL(1:2)/CNC(PVP)-10 (**b**, **e**); PHBV/PCL(1:2)/CNC(PVP)-15 (**c**, **f**). The scale is 2 μm.

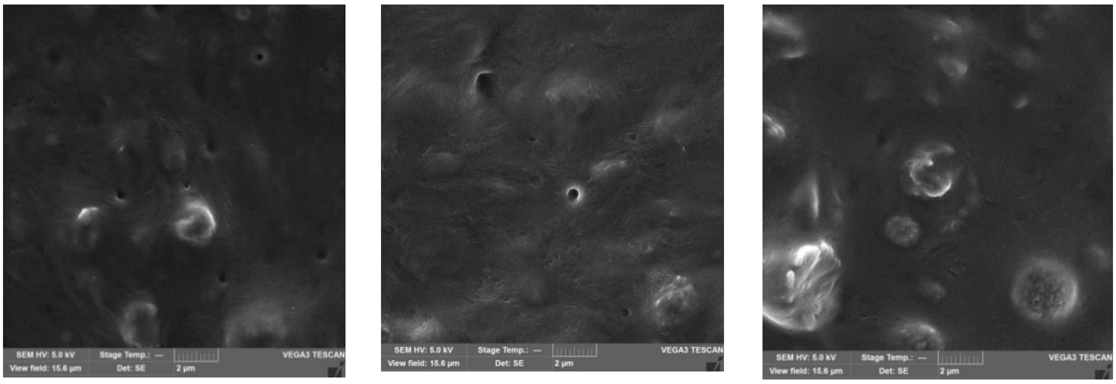

(**a**) (**b**) (**c**)

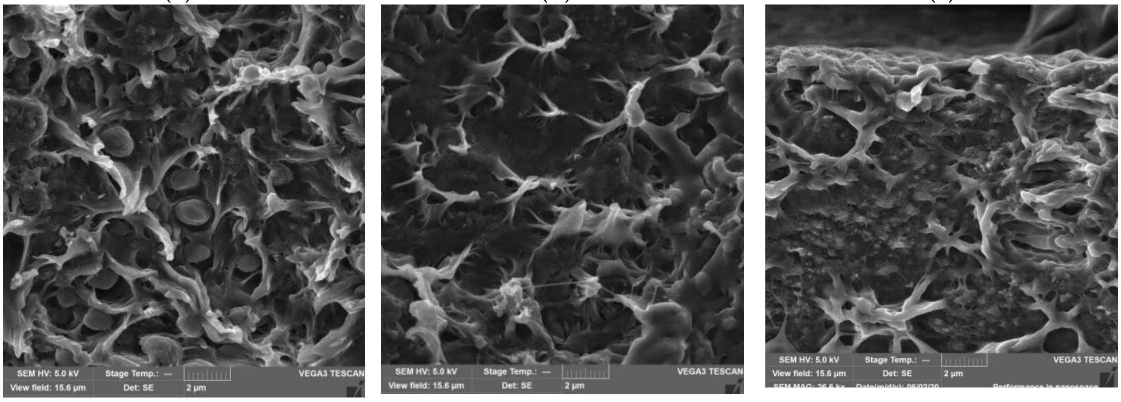

(**d**) (**e**) (**f**)

Figure S3. SEM images of the surface (a-c) and cleavage (**d**-**f**) of the PHBV/PCL/CNC composite films: PHBV/PCL(1:2)/CNC(PAM)-5 (**a**, **d**); PHBV/PCL(1:2)/CNC(PAM)-10 (**b**, **e**); PHBV/PCL(1:2)/CNC(PAM)-15 (**c**, **f**). The scale is 2 μm.

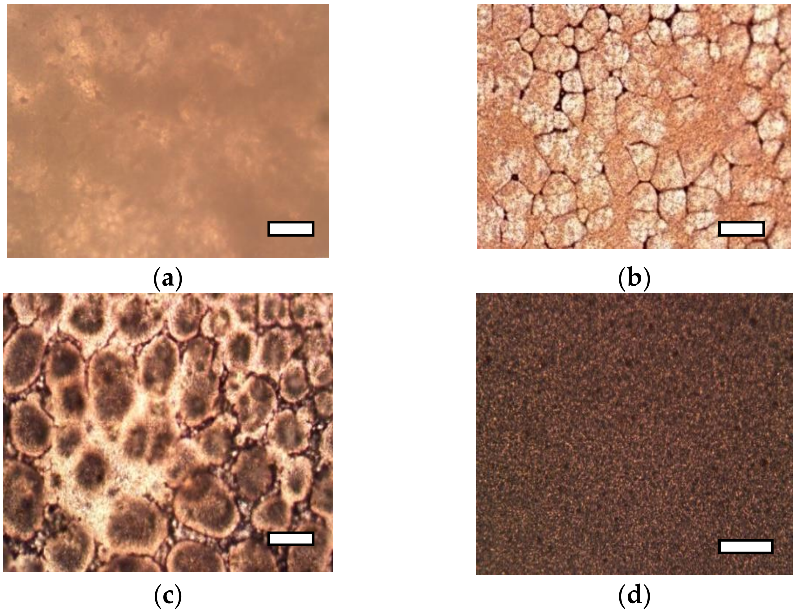

Figure S4. POM images of the surface of PHBV (**a**), PCL (**b**), and the PHBV/PCL mixture films: 1:1 (**c**); 1:2 (**d**). The scale is 100 μm.

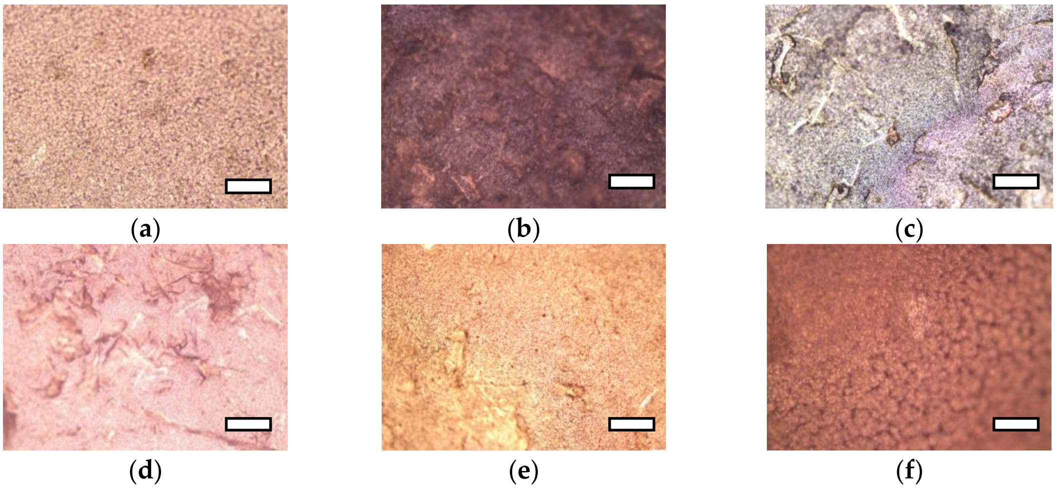

Figure S5. POM images of the surface of the PHBV/PCL/CNC composite films: PHBV/PCL(1:2)/CNC(PVP)-5 (**a**); PHBV/PCL(1:2)/CNC(PVP)-10 (**b**); PHBV/PCL(1:2)/CNC(PVP)-15 (**c**); PHBV/PCL(1:2)/CNC(PAM)-5 (**d**); PHBV/PCL(1:2)/CNC(PAM)-10 (**e**); PHBV/PCL(1:2)/CNC(PAM)-15 (**f**). The scale is 100 μm.

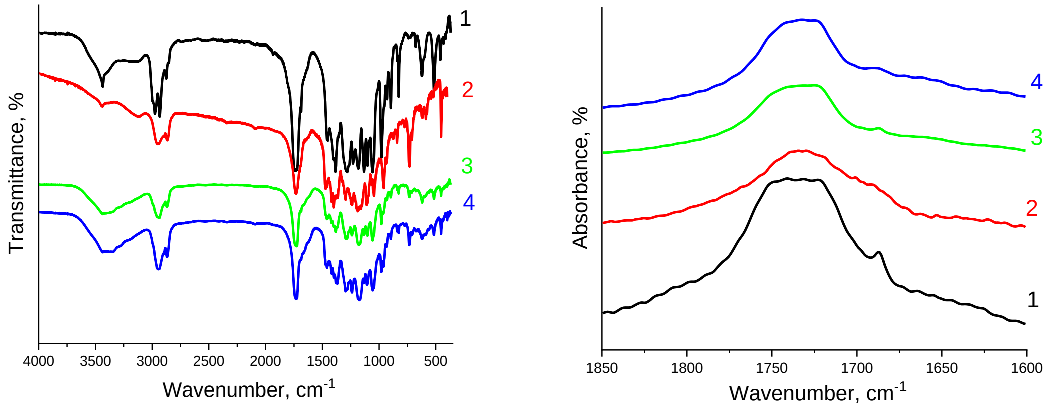

(a) (b)

Figure S6. FTIR spectra of the neat PHBV (1) and neat PLC (2), PHBV/PLC(1:1) (3) and PHBV/PLC(1:2) (4) mixtures: (**a**) in the 4000-400 cm^{-1} range; (**b**) in the 1850-1600 cm^{-1} range.

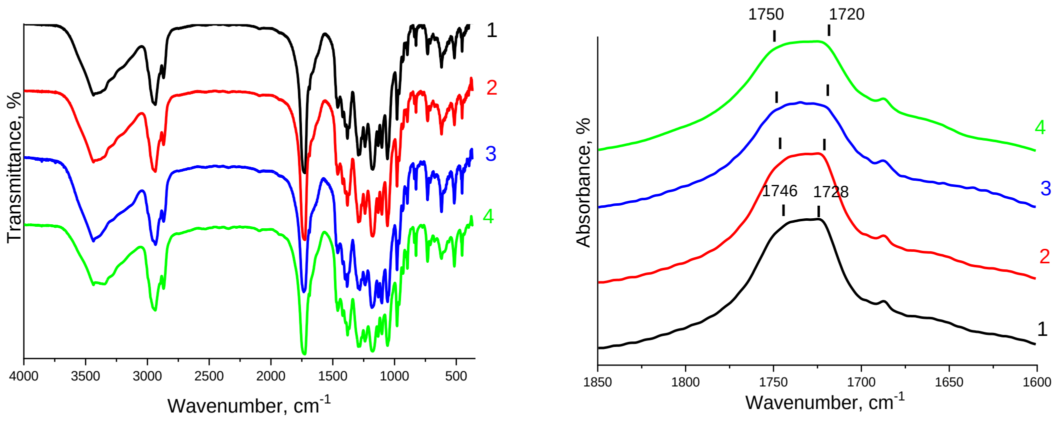

(a) (b)

Figure S7. FTIR spectra of the PHBV/PLC(1:1) mixture (1); PHBV/PCL(1:1)/CNC(PAM)-5 (2), PHBV/PCL(1:1)/CNC(PAM)-10 (3), PHBV/PCL(1:1)/CNC(PAM)-15 (4) composites: (**a**) in the 4000-400 cm^{-1} range; (**b**) in the 1850-1600 cm^{-1} range.

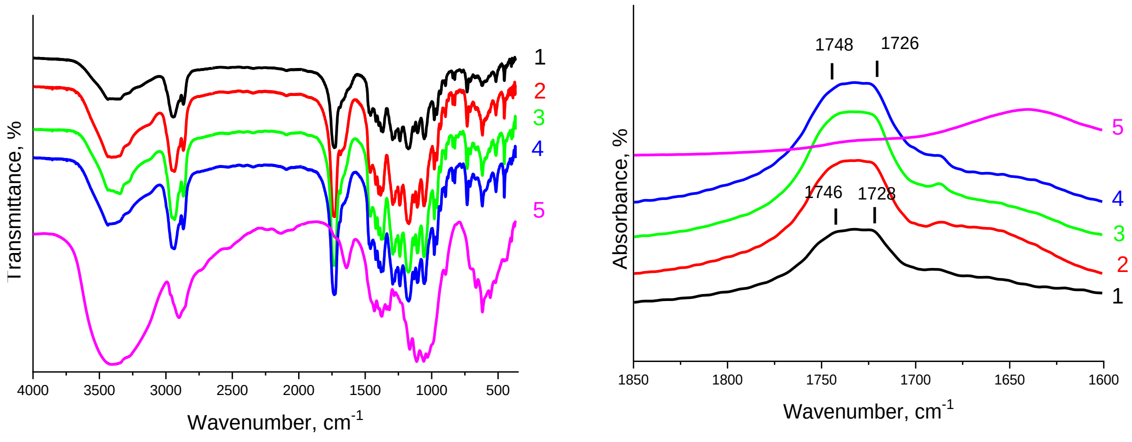

(a) (b)

Figure S8. FTIR spectra of the PHBV/PLC(1:2) mixture (1); PHBV/PCL(1:2)/CNC(PVP)-5 (2), PHBV/PCL(1:2)/CNC(PVP)-10 (3), PHBV/PCL(1:2)/CNC(PVP)-15 (4) composites; the neat CNC (5): (**a**) in the 4000-400 cm^{-1} range; (**b**) in the 1850-1600 cm^{-1} range.

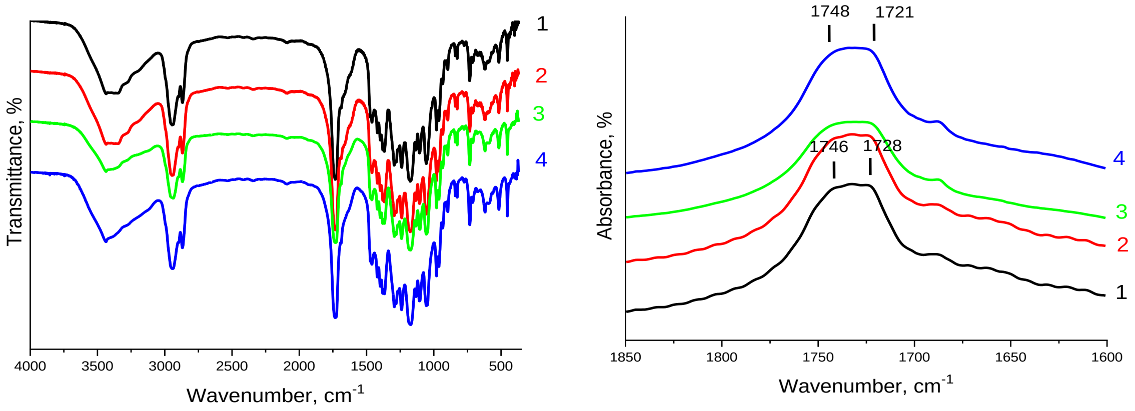

(**a**) (**b**)

Figure S9. FTIR spectra of the PHBV/PLC(1:2) mixture (1); PHBV/PCL(1:2)/CNC(PAM)-5 (2), PHBV/PCL(1:2)/CNC(PAM)-10 (3), PHBV/PCL(1:2)/CNC(PAM)-15 (4) composites: (**a**) in the 4000-400 cm^{-1} range; (**b**) in the 1850-1600 cm^{-1} range.

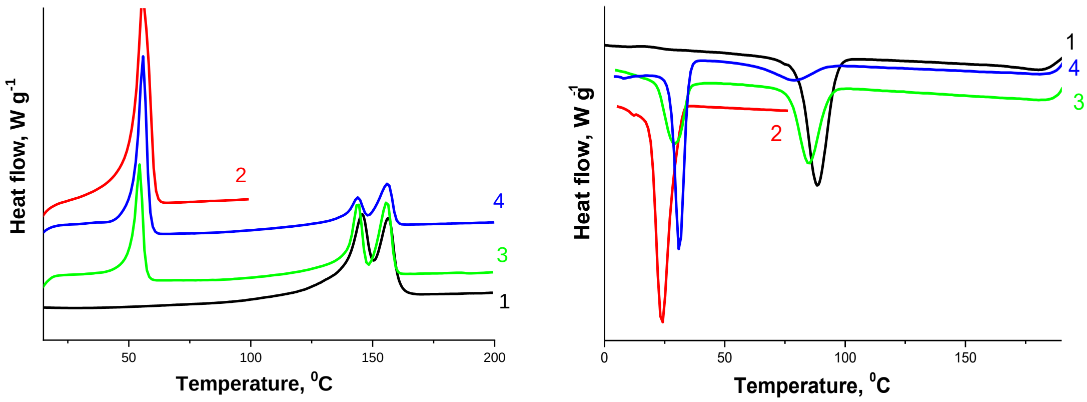

(**a**) (**b**)

Figure S10. DSC curves for neat PHBV (1), neat PCL (2); PHBV/PCL(1:1) (3), and PHBV/PCL(1:2) (4) mixtures: at heating (**a**); at cooling (**b**).

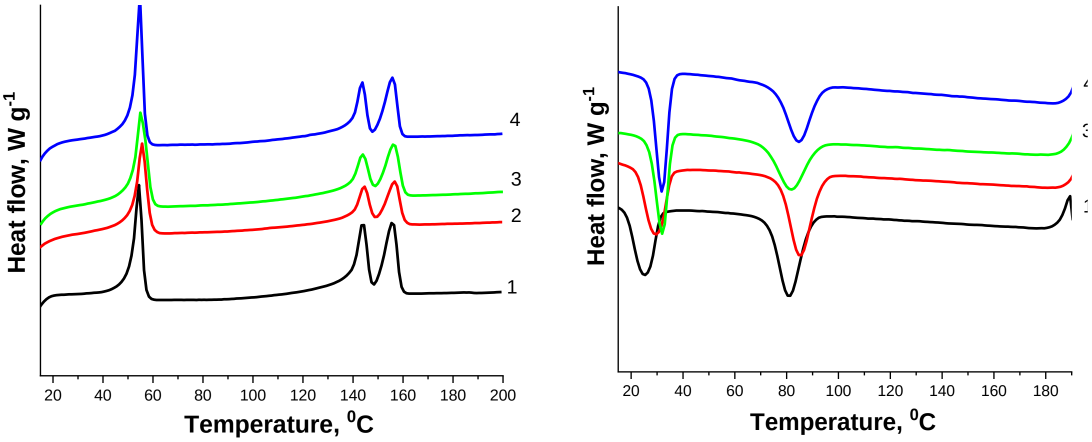

(**a**) (**b**)

Figure S11. DSC curves for the PHBV/PCL(1:1) mixture (1); PHBV/PCL(1:1)/CNC(PAM)-5 (2), PHBV/PCL(1:1)/CNC(PAM)-10 (3), and PHBV/PCL(1:1)/CNC(PAM)-15 (4) composites: at heating (**a**); at cooling (**b**).

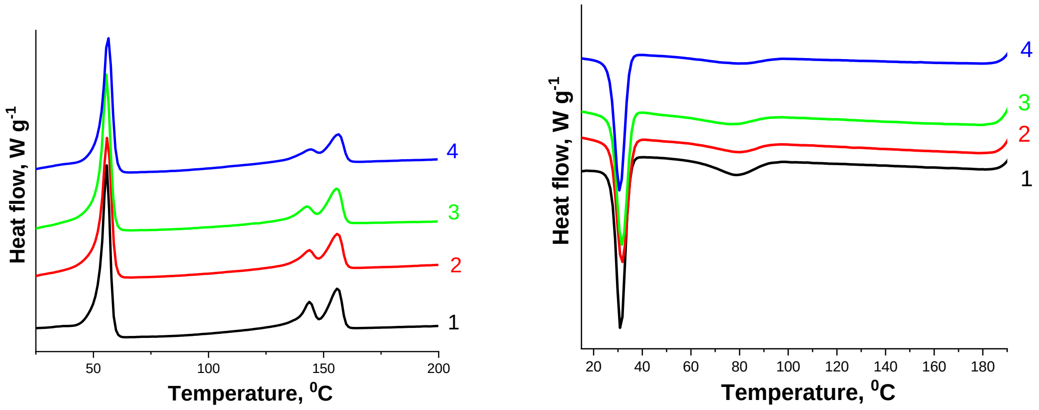

(**a**) (**b**)

Figure S12. DSC curves for the PHBV/PCL(1:2) mixture (1); PHBV/PCL(1:2)/CNC(PAM)-5 (2), PHBV/PCL(1:2)/CNC(PAM)-10 (3), and PHBV/PCL(1:2)/CNC(PAM)-15 (4) composites: at heating (**a**); at cooling (**b**).

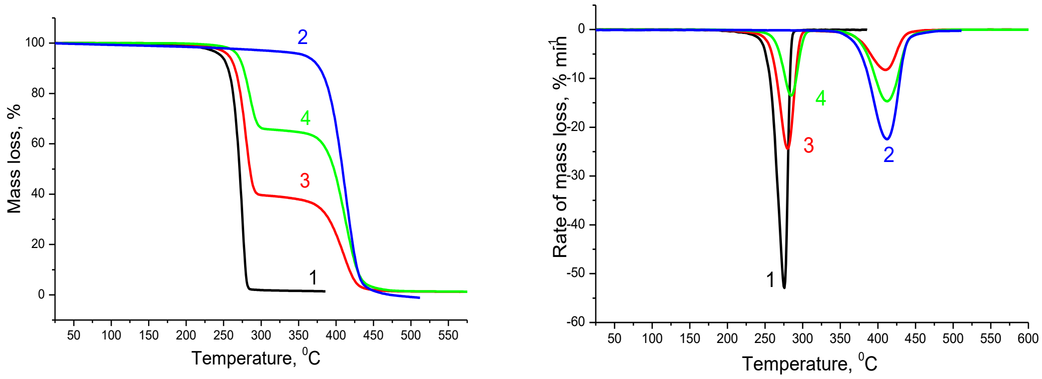

(**a**) (**b**)

Figure S13. TG (**a**) and DTG (**b**) curves for neat PHBV (1), neat PLC (2); PHBV/PCL(1:1) (3) and PHBV/PCL(1:2) (4) mixtures.

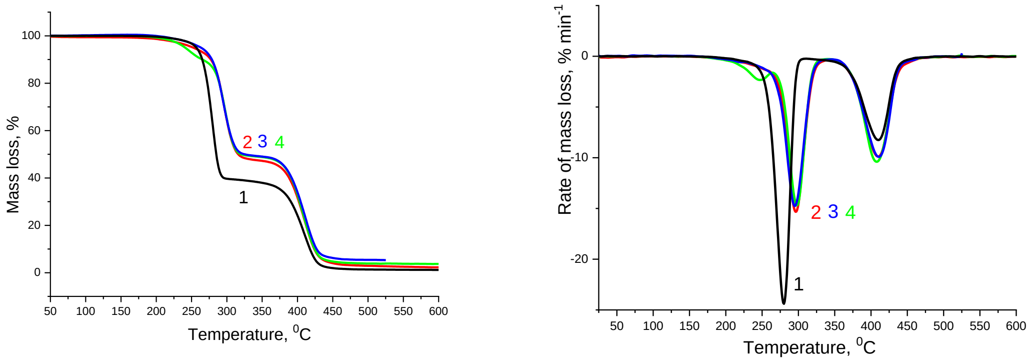

(**a**) (**b**)

Figure S14. TG (**a**) and DTG (**b**) curves for the PHBV/PCL(1:1) mixture (1); PHBV/PCL(1:1)/CNC(PVP)-5 (2), PHBV/PCL(1:1)/CNC(PVP)-10 (3), and PHBV/PCL(1:1)/CNC(PVP)-15 (4) composites.

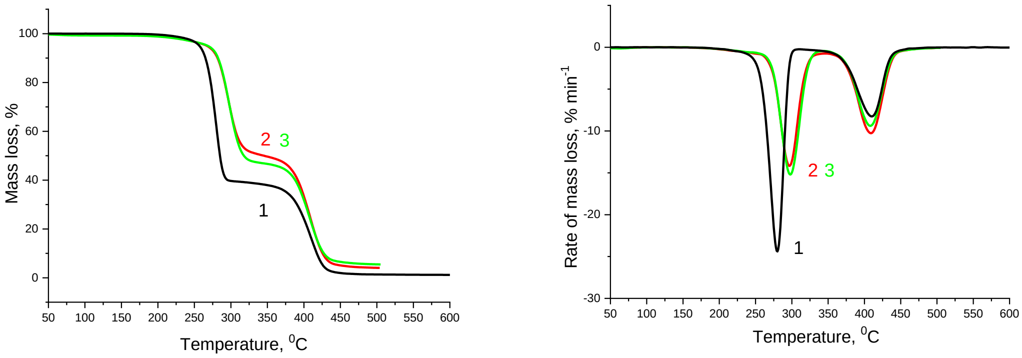

(**a**) (**b**)

Figure S15. TG (**a**) and DTG (**b**) curves for the PHBV/PCL(1:1) mixture (1); PHBV/PCL(1:1)/CNC(PAM)-5 (2), PHBV/PCL(1:1)/CNC(PAM)-15 (3) composites.

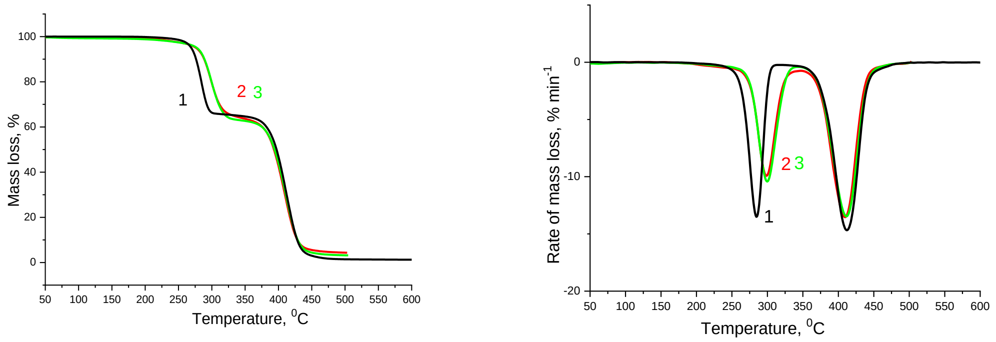

(**a**) (**b**)

Figure S16. TG (**a**) and DTG (**b**) curves for the PHBV/PCL(1:2) mixture (1); PHBV/PCL(1:2)/CNC(PAM)-5 (2), PHBV/PCL(1:2)/CNC(PAM)-15 (3) composites.

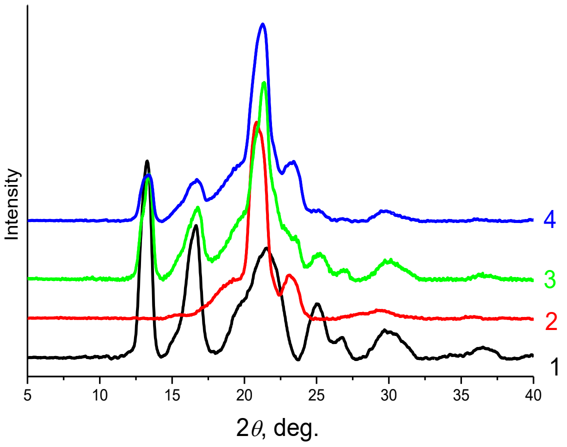

Figure S17. X-ray diffraction patterns of the neat PHBV (1), neat PCL (2); PHBV/PCL(1:1) (3) and PHBV/PCL(1:2) (4) mixtures

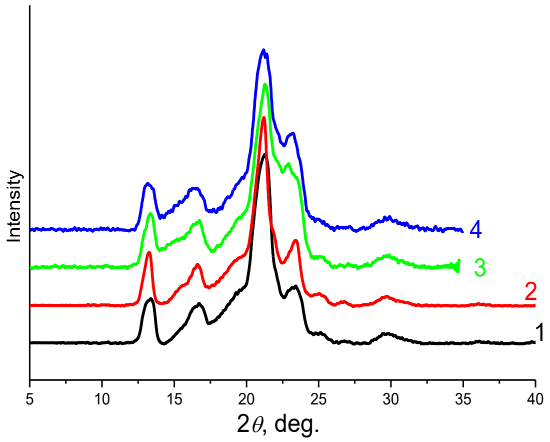

Figure S18. X-ray diffraction patterns of the PHBV/PCL(1:2) mixture (1); PHBV/PCL(1:2)/CNC(PVP)-5 (2), PHBV/PCL(1:2)/CNC(PVP)-10 (3), and PHBV/PCL(1:2)/CNC(PVP)-15 (4) composites.

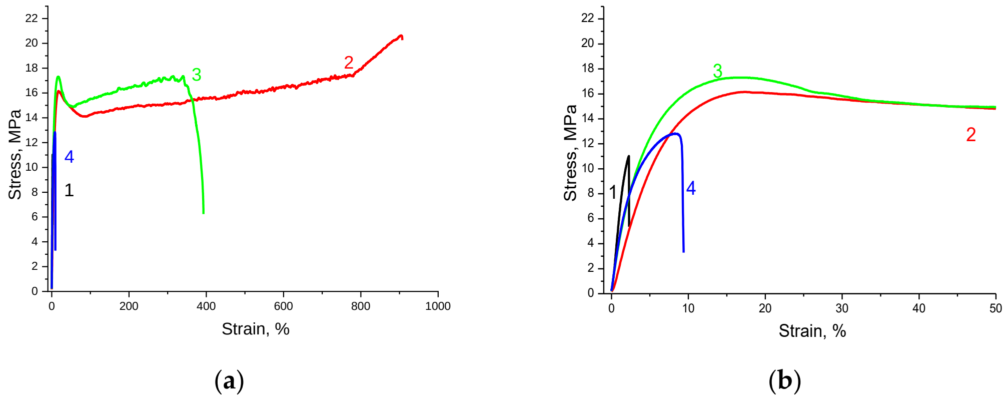

Figure S19. Stress-strain curves of the neat PHBV (1), neat PLC (2); PHBV/PCL(1:2) (3), and PHBV/PCL(1:1) (4) mixtures: (**a**) in the range of strain up to 1000%; (**b**) in the range of strain up to 50%.

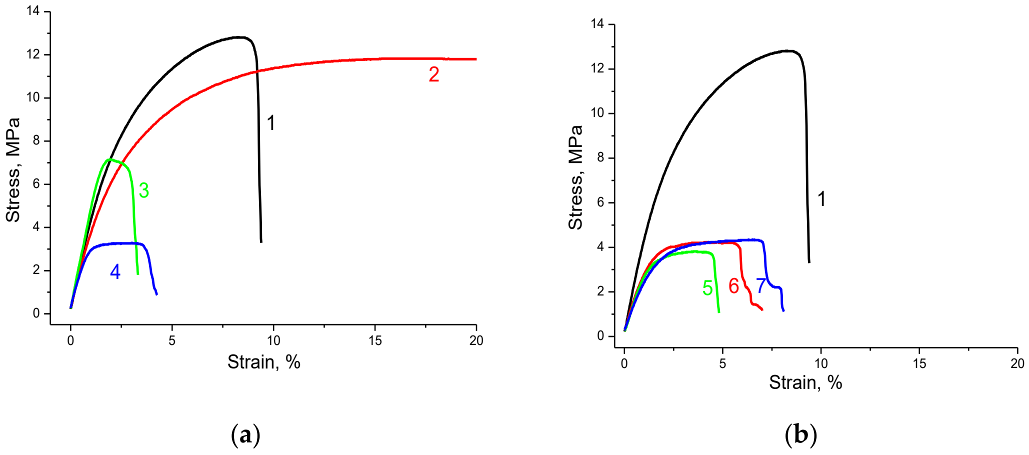

Figure S20. Stress-strain curves of the PHBV/PCL(1:1) mixture (1) and PHBV/PCL(1:1)/CNC(PVP)-5 (2), PHBV/PCL(1:1)/CNC(PVP)-10 (3), PHBV/PCL(1:1)/CNC(PVP)-15 (4) composites (**a**); PHBV/PCL(1:1)/CNC(PAM)-5 (5), PHBV/PCL(1:1)/CNC(PAM)-10 (6), and PHBV/PCL(1:1)/CNC(PAM)-15 (7) composites (**b**).

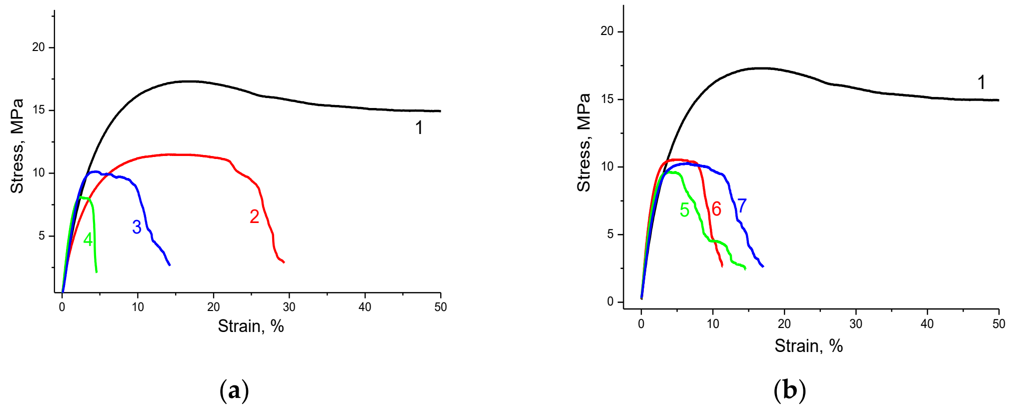

Figure S21. Stress-strain curves of the PHBV/PCL(1:2) mixture (1) and PHBV/PCL(1:2)/CNC(PVP)-5 (2), PHBV/PCL(1:2)/CNC(PVP)-10 (3), PHBV/PCL(1:2)/CNC(PVP)-15 (4) composites (**a**); PHBV/PCL(1:2)/CNC(PAM)-5 (5), PHBV/PCL(1:2)/CNC(PAM)-10 (6), and PHBV/PCL(1:2)/CNC(PAM)-15 (7) composites (**b**).
